# Network Strategies in Municipal Health Promotion: A Mixed‐Methods Study

**DOI:** 10.1002/hpm.70137

**Published:** 2026-09-16

**Authors:** Hanna Richter, Tatiana Mamontova, Eva Quante‐Brandt

**Affiliations:** ^1^ Department Health, Long‐term Care and Pensions University Bremen SOCIUM –Research Center on Inequality and Social Policy Bremen Germany

**Keywords:** community health, health equity, intersectoral collaboration, latent brokerage, network governance, network strategies

## Abstract

Municipal health promotion increasingly relies on intersectoral networks to address complex health challenges amid fragmented health, social and educational systems. However, little is known about how practitioners navigate these networks or how perceived strategies correspond with observed network structures. This convergent mixed‐methods study involved 14 health professionals and combined qualitative interviews with egocentric network analysis. Interview data were analysed using deductive content analysis, while the quantitative component comprised descriptive network analysis based on structural measures of connectivity, centrality and clustering. The qualitative findings show that network strategies are adaptive and situational, shaped by resource constraints, organisational routines, and network development rather than by explicit strategic plans. Bonding strengthened trust and existing relationship, clustering enabled task‐focused collaboration, formalisation supported continuity through established routines and bridging emerged largely unintentionally through routine coordination. Professionals combined strategies flexibly, resulting in hybrid strategic repertories rather than distinct strategy types. Integration of qualitative and quantitative data revealed only partial alignment between reported strategies and observed structures. Crucially, professionals themselves did not always recognise the structurally prominent brokerage positions they held, indicating that brokerage is an emergent feature of everyday networking. These findings suggest that municipal health promotion cannot be characterised by a single form of professional collaboration. Rather, collaboration must adapt to the task at hand, ranging from information exchange and multiprofessional collaboration to intersectoral collaboration. In order to sustain relationship‐based collaboration while retaining the flexibility to respond to local needs and health inequities, municipal health promotion requires institutional support for coordination.

## Highlights

- The combination of qualitative and egocentric network analysis reveals the differences between perceived strategies and network structure.
- Latent brokerage demonstrates that structurally significant network roles can emerge without any explicit strategic intent.
- Hybrid strategy configurations demonstrate how network practices adapt to changing coordination demands.

## 1 Introduction

The Ottawa Charter emphasises that health is a social and structural outcome shaped by living conditions, social status, and opportunities for participation. It therefore locates health promotion in the everyday settings in which people live, learn, work and participate [1]. As the level of government closest to citizens, municipalities are important settings for translating population health goals into local action. They can be understood as umbrella settings that link various everyday environments, such as schools, childcare facilities, workplaces and neighborhoods, to address social determinants of health and promote health equity [2, 3, 4].

However, the integrative role of municipalities is difficult to realize because responsibilities for health, social services, education and prevention are distributed across organisations with different mandates, resources and professional approaches. Persistent challenges such as chronic disease, unequal access to prevention and the consequences of pandemics have emphasised these structural gaps, increasing the need for local coordination [5, 6]. The Health in All Policies (HiAP) approach addresses this issue by emphasising the need for coordinated action across policy sectors and levels of governance. While HiAP provides the governance rationale for integrating health considerations into municipal policy‐making, it does not specify how intersectoral coordination is enacted in everyday practice [7, 8].

Intersectoral networks are therefore important instruments of municipal health promotion and local health governance, facilitating coordination, resource sharing and implementation of the Health in All Policies approach [9, 10, 11]. This is particularly relevant for professionals working in schools and neighborhoods, as they operate at the intersection of different settings and can facilitate connections between schools, municipal services, healthcare and community‐based support. Yet networks do not automatically generate integrated or sustainable collaboration. Their voluntary nature, diverse membership and limited institutionalisation mean that actors must negotiate relationships, coordinate interests and maintain collaboration over time [12, 13].

How these challenges are addressed has given rise to a variety of network‐based models and frameworks, including community coalitions, prevention chains and intersectoral coordination arrangements. These models differ in their organisational forms and contextual conditions, reflecting the need to adapt network arrangements to local circumstances [7, 14, 15]. Rather than seeking a single model that encompasses this diversity, this study focus on network strategies as relational practices through which actors address shared coordination challenges across different network arrangements. We examine four complementary strategies: *bonding*, *clustering*, *bridging*, and *formalisation*. Although they are conceptually distinct, they address complementary functions within network governance. *Bonding* strengthens existing relationships and supporting trust [16]. *Clustering* organises collaboration into functionally or thematically focused groups, helping to manage complexity [17, 18]. *Bridging* connects otherwise separated actors or sectors, facilitating access to information and resources [19, 20]. *Formalisation* consolidates collaboration through routines, agreements, or other institutional mechanisms, supporting continuity [21]. Together, these strategies provide a framework for analysing how municipal health promotion's integrative ambitions are realised through relationships.

This perspective distinguishes between relational practices and network structure. While network strategies describe how actors perceive and organise their network activities, network structure captures the observable patterns of relationships between those actors. As demonstrated by platform‐mediated governance [22], the effectiveness of networks depends on governance, structure, and relationships between actors. While these dimensions are related, they are not equivalent: strategies are enacted within existing relational and institutional contexts, while their structural implications depend on the wider network. However, there is limited empirical evidence on how such strategies are perceived and enacted in municipal health promotion. This study therefore links professionals' perceived network strategies to measurable characteristics of their observed networks, examining whether these practices are reflected in cross‐sectoral relationships.

## 2 Methods

The study employed a convergent mixed‐methods design, combining qualitative interview data and quantitative, ego‐centred network data collected from the same participants. These two components were analysed separately and then integrated through case‐based comparison to inform the overall interpretation.

The study provides a more nuanced understanding of how intentional actions translate, or fail to translate, into structural patterns of collaboration by integrating qualitative insights on actors' strategic reasoning with quantitative network‐analytic data. This methodological triangulation allows for the systematic exploration of subjective orientations and structural configurations, linking individual agency with network form.

### 2.1 Data Collection

The data is based on 14 semi‐structured interviews and corresponding ego‐centred network maps of healthcare professionals working in primary schools and in neighbourhoods in Germany. By focussing on multiple networks within one municipality, we were able to analyse structural differences under comparable institutional conditions. Germany is an important case study because cross‐sector health promotion involves fragmented governance, with responsibilities divided between autonomous organisations and municipalities that have discretion over local collaboration [3, 8]. Interorganisational networks therefore play a pivotal role in coordinating health‐related activities across boundaries.

During each interview session, participants first completed a semi‐structured interview, followed by ego‐centred network mapping. The sample size was determined based on the principles of purposive sampling and data saturation [23]. The sample was designed to provide sufficient diversity in terms of professional background, years of experience and field of activity, while ensuring that the volume of data was manageable enough to allow for in‐depth analysis. Participants were selected through an established collaboration, specifically because their roles involve actively building and managing professional networks in municipal health. The interviews, which were conducted between July 2023 and March 2024, lasted between 31 and 47 minutes. They were audio‐recorded and transcribed verbatim in accordance with the guidelines set out by Dresing and Pehl [24].

Network data were collected using Vennmaker, a free tool for visual network generation. As an ego‐centred design, each network reflects the actor's perspective on relevant relations. Following the concentric circle model of Kahn and Antonucci [25], respondents were asked to identify individuals, institutions, or task groups with whom they collaborate in their professional context. This resulted in 662 identified alters across all interviews. For each alter, additional attributes (e.g., organisational affiliation, role, or sector) and inter‐alter relations were recorded. This design enabled the examination of both the perceived relational structures and the network characteristics across cases [26]. Ethical approval was obtained from the University of Bremen, and all participants provided informed, written consent.

### 2.2 Qualitative Analysis

In this study, network strategies are not viewed as fully formalised or consciously planned interventions but rather as relational practices reflected in how professionals perceive and describe their network activities. A deductive approach was adopted for the qualitative analysis, with the four network strategies *bonding, clustering, bridging* and *formalisation* serving as analytical categories. The strategy categories were derived from their definitions in the network governance literature. Key characteristics were extracted from these definitions and used as sensitising criteria for coding the interview material. *Bonding* was thus associated with strengthening and maintaining existing relationships, trust, reciprocity and shared norms, while *clustering* was associated with dense, thematically or functionally focused groups and coordination. *Bridging* was associated with connecting otherwise separate groups and facilitating access to resources or information, and *formalisation* was associated with consolidating networks through formal structures or governance mechanisms [16, 17, 18, 19, 20, 21]. Rather than functioning as rigid keyword‐based rules, these characteristics guided the interpretation of relevant passages. Table S1 provides illustrative interview quotations, with key terms and characteristics derived from the literature highlighted in bold.

The transcripts were coded using MaxQDA 2020. While the initial aim was to assign one network strategy to each participant, iterative coding revealed that most interviews indicated more than one strategic orientation. All relevant passages were coded according to the four categories of the deductive strategy first, and the overall pattern within each case was assessed only once coding had been completed across all interviews. If one strategy was clearly more prominent, based on its recurrence and the clarity and strength of its arguments, it was identified as the predominant strategy. Where two strategies were equally prominent, the case was classified as a hybrid strategy. Therefore, the presence of multiple coded strategies within an interview did not automatically result in a hybrid classification. Any coding ambiguities were discussed among the researchers to enhance intersubjective consistency.

### 2.3 Ego‐Centred Network Analysis

The quantitative network analysis was both descriptive and exploratory. Network measures were employed to characterise structural configurations and inform their integration with qualitative strategy assignments. The analysis aimed to identify structural patterns within cases rather than test associations or causal relationships between network structure and strategic orientations, so no statistical inference or hypothesis testing was conducted.

The network data were processed and analysed with Gephi 1.0. For each ego‐centred network, descriptive network measures were calculated, including *network size*, *density*, *degree*, *betweenness centrality*, *harmonic closeness centrality*, *local clustering coefficient*, and *modularity*. Harmonic closeness centrality measures how accessible other actors are within the network. Meanwhile, the local clustering coefficient measures the extent to which an actor's immediate connections form additional connections with each other. The structural measures were interpreted using the study‐specific reference ranges and interpretative indicators presented in Table 1.

**TABLE 1:** Overview of network features, reference values, and strategic implications.

Network features	Measure level	Reference range^a^	Interpretation	Indicator for network strategies
Network size	Network	< 30 small; 30–50 moderate; > 50 large	Number of actors involved	Large networks favour bridging
Mean degree	Network	< 3 low; 3–7 moderate; > 7 high	Average connections per actor	High degree supports bonding/Formalisation
Edge density	Network	1.0–0.10 low; 0.10–0.30 moderate; > 0.30 high	Proportion of realised ties	High density = bonding; low = bridging
Average path length	Network	< 2.5 low; 2.5–4 moderate; > 4 high	Reachability across network	Short paths enable clustering/Bonding
Global clustering coefficient	Network	0.00–0.30 low; 0.30–0.60 moderate; > 0.60 high	Clique formation (standardised)	High values indicate clustering/Bonding
Modularity	Network	< 0.30 low; 0.30–0.70 moderate; > 0.70 high	Community structure (standardised)	High values signal clustering
Harmonic closeness	Node	< 0.30 low; 0.30–0.70 moderate; > 0.70 high	Node reachability, (standardised)	High values indicate bridging roles
Betweenness centrality	Node	< 0.10 low; 0.10–0.40 moderate; > 0.40 high	Brokerage role (standardised)	High values signal bridging
Local clustering coefficient	Node	0.00–0.30 low; 0.30–0.60 moderate; > 0.60 high	Closed neighbourhood (standardised)	High values indicate bonding
Number of triangles	Node	Few/medium/many (relative in network)	Triad participation	Many indicate bonding/Clustering

^a^ The reference ranges are based on empirical distributions within the investigated network. These values are therefore context‐specific and are intended to ensure consistent interpretation.

There is no generally accepted basis for threshold values that can be applied universally to ego‐centred network measures, as their interpretation depends substantially on the study design, the empirical context and whether ties are directed or undirected. Network size and density, in particular, can strongly influence the distribution of other network measures and therefore need to be considered when interpreting and comparing structural characteristics. In the present study, established social network analytic concepts guided the substantive direction of interpretation, whereas the specific ranges and categories in Table 1 were derived from the empirical distribution of the measures across cases, considering the relational configurations reported by the focal actors in the egocentric networks [27, 28].

The calculated network measures capture different aspects of network structure, which were then interpreted in relation to the strategies under study. For example, network size indicates relational reach, while density and degree describe the extent of connectivity. Betweenness centrality indicates brokerage between otherwise less‐connected parts of the network, and harmonic closeness reflects the accessibility of other actors within the network. The local clustering coefficient captures the interconnectedness of an actor's immediate contacts, and modularity indicates the extent to which the network contains differentiated subgroups [27]. These indicators were therefore used to characterise structural tendencies, which could then be compared with the strategies identified through qualitative analysis. They were not used as direct measures of the strategies themselves. Where structural indicators pointed towards more than one strategy, the network was classified as reflecting a hybrid strategy. This is analogous to the qualitative classification of cases in which two strategic orientations were identified.

### 2.4 Case‐Level Integration of Qualitative and Quantitative Findings

Integration was conducted at case level by comparing the strategies identified in the qualitative analysis with the structural strategy indicators derived from the corresponding ego‐centred network. Hybrid strategies were retained during case‐level integration. Qualitative and quantitative classifications were considered to be convergent if the identified strategies were consistent across both data sources. Partial convergence was assigned when the two data sources shared at least one strategy. Divergence was assigned when the qualitative and quantitative classifications indicated different strategic orientations with no corresponding overlap. Thus, the alignment assessment considered the overall pattern of indicators rather than requiring a one‐to‐one correspondence between individual network measures and strategies.

The resulting case‐level classifications were based on integrating qualitative strategy assignments with quantitative network characteristics. This approach allowed us to compare perceived strategic orientations with structural network characteristics while acknowledging that qualitative and quantitative data may capture different aspects of networking practice.

## 3 Results

### 3.1 Subjective Strategy Descriptions

The analysis of the qualitative data revealed that all participants described networking‐related practices aimed at strengthening cooperation within municipal health promotion structures. These practices were rarely described in terms of explicit strategies. Instead, participants described practical courses of action shaped by how they interpreted problems, mobilised relationships and navigated the contextual demands of intersectoral collaboration. All four strategies identified in network research were reflected in the interviews, although participants often combined several strategies or used different strategies depending on the situation.

The majority of participants described that a 'trusting and appreciative atmosphere' (P5, para. 42), combined with continuity, as necessary in order to build a network ‘that you can rely on’ (P6 para. 92). These descriptions suggest that *bonding strategies* were considered important for establishing and maintain relationships. Health professionals reported that these practices helped them navigate the initially diverse and complex landscape of stakeholders in municipal health promotion. One professional explained, ‘I think a network is something you have to build and something you have to maintain’ (P13, para. 2). This illustrates how participants described *bonding* as active relational work rather than a passive by‐product of cooperation.

*Clustering strategies* were described by actors who coordinated activities within thematic or task‐specific subgroups. These configurations were often described in response to identified needs, with professionals intentionally mobilising and assembling the necessary expertise to fulfil those requirements. One participant articulated this clearly: ‘We had this specific need, and then we looked at who else was out there, who had expertise or experience. Which person could we bring in? And then we contacted them’ (P9, para. 24). C*lustering* is therefore considered a form of problem‐driven mobilisation, rooted in professionals' interpretations of needs and their ability to convene actors. Cluster‐based cooperation was perceived as more efficient and purposeful, enabling professionals to ‘work together more effectively’ (P2, para. 8) These clusters were often used to align activities across different administrative or organisational units, such as linking neighbourhood projects with educational settings.

*Formalisation strategies* were outlined as ways of consolidating cooperation by means of structured meetings, documentation practices and formal agreements. These structures were valued not only for creating clarity and continuity but also for making collaboration less dependent on individual staff. As one interviewee explained, ‘I joined this network, and there was already something before me, and there will also be something after me. I don't have to reinvent the wheel… using structures that already exist makes work processes faster’ (P10, para. 4). Participants primarily described *formalisation* in relation to more established phases of network development. They explained that structured routines, such as regular exchange, provided stability and ‘created a team feeling’ (P6, para. 4) over time.

Unlike the other strategies, *bridging* was rarely mentioned as an intentional approach. Only one healthcare professional described the deliberate connection of people to enable joint activities. They explained, 'the contacts I have… helped them carry out their own project' (P14, para. 46). The majority of respondents did not consider the networking of individuals to be a planned strategy. Instead, participants identified *bridging* as an unintended consequence of everyday coordination work involved in collaboration across sectors, such as health, education, and social services. *Bridging* was most often described from the perspective of benefiting from the contacts of network partners, rather than actively brokering new connections themselves.

### 3.2 Structural Network Characteristics

The egocentric network analysis provided complementary insights into the structural configurations of municipal health promotion networks (see Table 2). While the *network size* ranged between 29 and 64 people, the majority showed moderate *average degree* values. This indicates that most actors maintained direct connections with a moderate number of partners. The networks therefore demonstrated a certain degree of inter‐organisational exchange despite limited connectivity. The low *edge density* observed in all cases reflects the relatively limited connectivity reported among alters within the egocentric networks.

**TABLE 2:** Network characteristics of ego‐centred networks.

Participant	Network size	Average degree	Edge density	Average path length	Global clustering coefficient	Modularity	Harmonic closeness	Betweenness centrality	Local clustering coefficient	Number of triangles
Ego 1	53	*4.830*	*0.093*	2.495	0.424	*0.373*	0.814	0.437	*0.033*	11
Ego 2	49	6.776	0.141	2.715	0.451	0.445	*0.670*	0.379	0.132	18
Ego 3	*41*	8.293	0.207	2.761	*0.375*	0.417	*0.609*	*0.278*	0.054	*5*
Ego 4	38	6.000	0.162	*2.462*	0.656	0.452	0.796	0.539	0.105	18
Ego 5	40	**11.800**	**0.303**	*2.073*	**0.716**	0.419	**0.900**	0.495	**0.174**	**66**
Ego 6	42	7.330	0.179	2.281	0.517	0.423	0.852	0.523	0.080	11
Ego 7	*38*	6.526	0.176	2.424	0.596	0.485	0.727	0.317	0.120	11
Ego 8	44	5.591	0.130	2.496	**0.717**	**0.579**	0.833	**0.762**	*0.030*	10
Ego 9	61	7.639	0.127	2.468	0.647	0.476	0.815	0.497	0.062	29
Ego 10	45	*5.067*	0.115	**2.923**	0.397	0.455	0.707	0.458	0.065	*10*
Ego 11	29	6.897	**0.246**	2.723	0.556	**0.482**	0.676	0.365	**0.218**	12
Ego 12	55	**10.291**	0.191	2.315	0.539	*0.405*	**0.863**	**0.550**	0.107	**64**
Ego 13	**64**	6.321	*0.100*	2.678	0.438	0.433	0.680	*0.241*	0.080	18
Ego 14	**63**	8.762	0.141	**2.788**	*0.283*	0.444	0.700	0.403	0.116	35

*Note*: Bold type indicates the highest value in the table; italic type indicates the lowest value.

Nevertheless, clear structural patterns emerged. Firstly, there were strong differences in clustering‐related features: global *clustering coefficients* ranged from 0.28 to over 0.71. High clustering values, particularly for participants 5, 8 and 9, point to locally cohesive subnetworks, and were interpreted as consistent with the *clustering* strategy. Second, several networks exhibited structural profiles that were interpreted as consistent with *formalisation* strategies. Networks with low *modularity*, higher *harmonic closeness*, and comparatively even *degree distributions*, such as those of Participants 1 and 12, showed more integrated and evenly distributed relational structures. Third, structural characteristics consistent with *bridging* appeared in a smaller subset of cases. High *betweenness centrality* values indicated particularly prominent structural bridging positions for Participants 5 and 8. In other cases, intermediate betweenness scores, such as for Participants 6 and 9, indicated less pronounced *bridging* positions, often in combination with *clustering* indicators. This means that structural *bridging* often coexisted with other structural features rather than constituting an isolated structural pattern.

Across the sample, structural metrics frequently pointed towards hybrid constellations rather than a single predominant structural pattern. For example, Participant 5 showed structural characteristics consistent with both strong *clustering* and *bonding* tendencies. Participant 6, as previously mentioned, showed structural characteristics consistent with a combination of *bridging* and *clustering*, while Participant 12 showed structural characteristics consistent with both *formalisation* and *bonding*. In contrast, a small number of networks (e.g., Participants 3, 10, 14) displayed structural configurations with weak or inconsistent indicators, making an unambiguous classification difficult.

Overall, the networks exhibited varying degrees of connectivity, clustering and brokerage, with several cases displaying a combination of these structural characteristics. These characteristics allowed us to identify cases in which the observed network structure was interpreted as consistent with one predominant strategy, as well as hybrid cases in which structural characteristics were consistent with two strategies.

### 3.3 Triangulation of Strategies and Structures

The comparison of professionals described strategies with measured network characteristics revealed partial alignment but also frequent discrepancies (see Table 3). These findings suggest that, while network strategies express professionals' agency, they are shaped by the structural conditions of the network in which professionals operate.

**TABLE 3:** Triangulation of qualitative strategies (from interviews) and quantitative strategies (from network analysis).

Participant	Qualitative strategy (interviews)	Quantitative strategy (network analysis)	Alignment status^a^
Ego 1	Formalisation/Bonding	Formalisation	Partial convergence (−)
Ego 2	Bridging/Bonding	Bonding/Bridging	Convergence (+)
Ego 3	Need‐oriented practice/Bonding	Bonding	Partial convergence (−)
Ego 4	Formalisation	Bridging/Formalisation	Partial convergence (−)
Ego 5	Formalisation/Bonding	Clustering/Bonding	Partial convergence (−)
Ego 6	Bonding/Formalisation	Bridging/Clustering	Divergence (!)
Ego 7	Clustering/Bonding	Clustering	Partial convergence (−)
Ego 8	Clustering/Bonding	Bridging	Divergence (!)
Ego 9	Clustering	Bridging/Clustering	Partial convergence (−)
Ego 10	Bonding	Bonding	Convergence (+)
Ego 11	Formalisation	Clustering	Divergence (!)
Ego 12	Formalisation/Bonding	Formalisation/Bonding	Convergence (+)
Ego 13	Need‐oriented practice/Bonding	Bonding	Partial convergence (−)
Ego 14	Bonding/Bridging	Formalisation	Divergence (!)

^a^ Meaning of the symbols: (+) = Convergence; (−) = partial convergence; (!) = Divergence.

In several cases, the described strategies corresponded to structural characteristics. Participant 5, for instance, showed a high *clustering coefficient* (0.716) and a large *triangle count* (66), reflecting a robust, locally interconnected structure. Participant 9 likewise displayed pronounced structural *clustering* (0.647) alongside moderate *modularity* (0.476), aligning with their qualitative account of managing smaller task‐focused groups. Convergence was also observable for participants whose narratives emphasised more formalised coordination. Participants 1 and 12, for example, exhibited structural features that were interpreted as consistent with the *formalisation*, including lower *modularity*, high *harmonic closeness*, and stable *degree* distributions, mirroring their descriptions of structured routines and regulated collaboration. Partial convergence occurred where structural characteristics were consistent with more than one strategy. For example, Participant 4 was classified as hybrid case combining *formalisation* with partial *bridging*. This corresponded with their qualitative account of working across administrative boundaries. Similar hybrid patterns emerged for participants 6 and 12, whose measured network characteristics were interpreted as consistent with combinations of *bridging* and *clustering*, and *bonding* and *formalisation*, respectively.

Several cases, however, revealed discrepancies between described strategies and measured network characteristics, offering insights into the limits of self‐reported strategy. Participant 8 stands out most clearly: their network showed the strongest structural bridging position in the sample, yet their qualitative narrative emphasised formalisation and clustering rather than brokerage. This discrepancy illustrates that structurally prominent brokerage positions may not be reflected in professionals' own descriptions of their networking strategies. Deviations primarily occurred in cases with weak or inconsistent structural indicators, as well as in cases where the qualitative strategy descriptions did not correspond to the structural characteristics interpreted as consistent with particular strategies. Participant 3's needs‐oriented approach in the qualitative data contrasted with their structural configuration, which showed the lowest clustering coefficient and lowest modularity in the sample.

## 4 Discussion

The findings support a network‐based perspective across different forms of municipal health promotion networks. Although community coalitions, prevention chains and intersectoral coordination arrangements differ in organisational form [15], participants described *bonding*, *clustering*, *bridging* and *formalisation* as relational practices that address similar coordination challenges. The combination of these practices varies according to local demands and contexts.

The discrepancy between intentional action and observed relational positions is evident in several cases, most notably in Case 8. While the health professional described their role primarily in terms of formal coordination, network analysis revealed that they occupied a structurally central brokerage position which was not reflected in their own description of the role. This illustrates how the cumulative effects of everyday networking practices may extend beyond the strategic awareness of the actors involved. Although few health professionals explicitly described *bridging* as an intentional strategy, network data revealed structurally central brokerage roles (see Table 3). These can be conceptualised as *latent brokerage* positions, in which actors occupy structurally important intermediary roles without explicitly describing brokerage as part of their networking strategy. The findings suggest that brokerage should be understood not only as an intentional networking strategy, but also as an emergent outcome of everyday interaction.

Comparing perceived strategies with observed network structures provides a more nuanced understanding of network governance. While qualitative accounts capture how professionals understand and enact their networking practices, network analysis reveals relational configurations that may extend beyond individual perceptions. This perspective extends existing research that typically treats network strategies as distinct and stable approaches. Although concepts such as weak versus strong ties [19] and formalised versus participant‐governed coordination [21] are useful for distinguishing between different relational and governance logics, the findings suggest that these logics can coexist within the same network. This implies that municipal health promotion cannot be characterised by a single form of professional collaboration. Rather, depending on the task at hand, different relational practices may be activated, ranging from information exchange and multiprofessional collaboration to intersectoral collaboration, while being shaped by organisational routines, available resources, fragmented governance arrangements, and the developmental stage of the network. Consequently, network strategies can be understood less as fixed properties of actors or network types, and more as flexible repertoires of relational practices shaped by professional agency and structural conditions.

The agency–structure dynamics have specific implications for health equity in municipal health promotion. Beyond micro‐level strategy–structure interactions, municipal health promotion networks are fulfiling compensatory functions within fragmented health and welfare systems. From a health equity perspective, these functions are not neutral. They are particularly relevant for populations facing structural disadvantages and limited access to coordinated care. Schools are an ideal setting in which to fulfil this compensatory function. In Germany, compulsory school attendance provides regular contact with children across social groups, making schools an accessible setting for identifying health and social needs. This accessibility is particularly relevant for children and families with limited engagement with the healthcare system. This enables health professionals to identify needs at an early stage, build trusting relationships and potentially reduce access barriers to health and social services for children and families who might otherwise be underserved. Consequently, health professionals working in community and school‐based settings bridge the gaps between healthcare, social services, education, and family context.

However, relationship‐based networking remains vulnerable to staff turnover, the capacities of individuals, and local resource constraints. A strong reliance on individual relationships can undermine health equity when access to support and its continuity depend on personal capacities and discretionary engagement rather than institutional embedding [29]. Although setting‐based approaches can improve equitable access, they require stable institutional structures to ensure long‐term sustainability and address the structural causes of health inequalities. This vulnerability underscores the importance of institutionalising coordination without replacing the relational nature of networking. While formalised routines can provide continuity when individual actors, political priorities or resources change, relational strategies remain necessary to adapt collaboration to local needs. Therefore, institutionalised coordination can stabilise relational capacities, reducing dependence on individual actors while preserving the flexibility required for context‐sensitive collaboration.

While the findings do not point to a universal model for municipal networking, they do provide several orientations for network development that could be applicable beyond the specific context studied. First, relational depth should be established as networks develop and expand. Bonding strategies support continuity amid high staff turnover and limited resources while providing important access points to networks for smaller civil society organisations and marginalised groups [9, 10]. Second, functional diversity can help manage complexity without excessive formalisation. Creating thematic subgroups can be particularly relevant for interwoven challenges such as chronic diseases, social exclusion and mental health, which require cross‐sectoral cooperation [22]. Third, the formalisation of institutional routines can strengthen network continuity in the face of political fluctuations and resource constraints in decentralised governance systems [6]. At the same time, the diversity of local network arrangements means that shared reference points are important for reflecting on network development. Standardised, small‐area health data could provide this basis by supporting local needs assessments, transparency, and comparisons across settings while enabling network responses to remain context‐sensitive [30, 31]. Overall, these findings suggest that resilient municipal health governance depends on balancing trust‐based relationships, manageable complexity, institutional continuity and shared informational foundations.

The findings also indicate that municipal health promotion networks can be understood as adaptive, socially negotiated systems, in which relational functions emerge through the interaction of professional agency and structural conditions. Latent brokerage demonstrates how important network functions can develop without any explicit strategic intent, and the coexistence of different relational logics shows that network coordination cannot be reduced to a single, stable strategy or network type.

From a research perspective, future studies should examine how network strategies evolve over time, and how policy changes and funding structures reshape network configurations. Longitudinal mixed‐methods designs appear promising for revealing whether intentional adaptation of strategies leads to measurable improvements in cooperation and health equity. Further research could examine whether latent brokerage positions emerge, persist or change as networks develop and how institutionalisation affects the relationship between perceived strategies and structural network characteristics.

## 5 Limitation

This study has several limitations that should be considered when interpreting the findings. Firstly, the sample size was relatively small, primarily due to the time‐consuming nature of data collection, and comprised a specific group of health professionals working in municipal settings, all of whom were based in Bremen. This allowed for in‐depth, context‐sensitive insights, but the geographical and sectoral concentration of the sample limits the diversity of municipal contexts represented in the study. The findings should therefore not be interpreted as statistically generalisable across municipalities, and their transferability to other municipal contexts remains to be examined. Furthermore, as participation in the study was voluntary, there is potential for selection bias; it is possible that more engaged or reflective professionals were over‐represented, which may have influenced the observed patterns. Secondly, as the study relied on self‐reported interview data, participants may have described their networking practices in a way that was socially desirable. This could have led to an overemphasis on strategic or collaborative behaviours. Thirdly, the data are cross‐sectional, capturing network structures and strategies at one point in time. Consequently, the study cannot account for the dynamic and evolving nature of networks, where strategies and relationships may shift in response to organisational or social contexts.

Additionally, the methodological limitations inherent to qualitative research and ego‐centred network analysis must be acknowledged. Although the qualitative analysis was theory‐driven, the coding and interpretation processes are still interpretive and cannot be fully standardised. Similarly, ego‐centred network analysis relies solely on the interviewee's perspective; the perspectives of other network members are not considered. However, prior research has shown that the validity of network data with name generation is relatively high [32].

Finally, while triangulation between qualitative and quantitative data enhances robustness, the operationalisation of strategies in network metrics remains a conceptual approximation. Future research should further refine the conceptual and methodological tools linking agency and structure, possibly through longitudinal or comparative mixed‐methods designs.

## 6 Conclusion

This study emphasises the importance of viewing municipal health promotion networks as dynamic systems of action. By integrating qualitative insights with egocentric network analysis, the study shows that professionals' strategies are adaptive, context‐sensitive practices shaped by resource constraints, organisational routines, and local governance demands, rather than fixed orientations. The finding that individuals combine multiple strategy types reveals coexisting relational practices that are activated in specific situations. Meanwhile, the partial alignment between reported strategies and observed network structures highlights the distinction between intentional action and emergent structural effects. Notably, professionals did not always recognise their own structurally prominent brokerage positions as strategic roles. These findings highlight how hybrid configurations and latent brokerage can emerge beyond actors' explicit strategic intentions.

From a practical perspective, the results emphasise the importance of cultivating relational infrastructures for effective and equitable health promotion, while also revealing the limits of networks as compensatory mechanisms. Although networks currently compensate for systemic fragmentation, a sustained reliance on individual professionals and project‐based arrangements constrains their transformative potential. Therefore, strengthening municipal health promotion requires stronger institutional support for coordination that enable local public health actors to systematically address preventive care gaps and health inequalities in the long term. This demands a balance between institutional continuity and the relational and context‐sensitive character of networking.

From a theoretical perspective, the study advances the idea that networks are adaptive, socially negotiated systems rather than static structures. Future research should use longitudinal and comparative methods to examine how strategies and network structures evolve over time and how changes in context reshape their relationship.

## Author Contributions

Hanna Richter conceived and designed the study, led the collection of data, conducted the main analyses, and wrote the first draft of the manuscript. Tatiana Mamontova contributed to the analysis of the data, including qualitative coding, and provided support with the egocentric network analysis. Professor Quante‐Brandt provided methodological and conceptual guidance throughout the research process, contributing substantially to the revision and refinement of the manuscript. All authors reviewed and approved the final version of the manuscript.

## Funding

The authors have nothing to report.

## Ethics Statement

Ethical clearance was obtained from the ethics committee of the University of Bremen to conduct the research (reference number 2023‐17). An informed written declaration of consent is signed by all study participants. The respondents' right to refuse or withdraw from participating in the interview at any time was fully respected, and the information provided by each respondent was kept confidential by making each transcript coded and not sharing personal information of any patient of the third party.

## Conflicts of Interest

The authors declare no conflicts of interest.

## Supporting information


**Table S1:** Illustrative Interview Quotes and Corresponding Network Strategies.

## Data Availability

The data generated and analyzed during this study are subject to ethical and data protection restrictions and are therefore not publicly available. The corresponding author can provide fully anonymized transcripts and adjacency matrices without any node‐level attributes upon reasonable request for scientific purposes. This is provided that data sharing is compatible with the original consent of the participants or that additional consent has been obtained where necessary.
