# Supplementary material for: Network Strategies in Municipal Health Promotion: A Mixed‐Methods Study
Source: Int J Health Plann Manage. 2026 Sep 16;41(6):1340–9. doi: 10.1002/hpm.70137 (PMC13730245; doi:10.1002/hpm.70137)
Supplement: Supplementary file 1 [file HPM-41-1340-s001.docx]

| Participants | Example sentence | Network category |
| --- | --- | --- |
| Ego 1 | Perhaps even **to connect certain people** (..) to certain networks or **institutions in order to (..) yes (..) work together** in a health-promoting and efficient manner. | Bridging |
|  | And above all, it's low-threshold (..) because if you use familiar actors (..) then the shyness isn't so high (..) because a certain (..) yes, **a certain relationship is already there**. | Bonding |
|  | I also see the effect of my network when, for example, **I am approached by (…) politicians** or whatever, so that I am noticed. | Formalization |
| Ego 2 | Um, these are the **opportunities** offered by the local community, for example, from various institutions, which students **wouldn't otherwise have access to**. Through this networking, these opportunities can take place and children can participate in activities they would never have heard of before. | Bridging |
|  | Uh, I have some really GREAT contacts there, lots I can actually turn to, call, and I know it'll be **sorted out within an** **hour**. People I can simply rely on. | Bonding |
|  | Um (..), there's also a certain regularity to it. It's not like you just say, "We've been in touch, right?" and then it's the same thing again. When you meet regularly and regularly plan activities together, a feeling develops that you can **rely** **on someone** and that you're (..) not a team, but definitely a sense of camaraderie. | Formalization and Bonding |
| Ego 3 | **Without my network, I wouldn't be able to act,** I wouldn't be able to learn about or compare needs, and I would lack feedback. | Need-oriented Practice |
|  | So it was more like this: either the institutions already had a **concrete idea or questions that they approached me** with, OR I, for example, contacted the family center because I saw **potential for collaboration** there, and they said, "Just come to the parents' café." | Need-oriented Practice |
|  | Everyone needs to be **reliable**, and the **tasks need to be distributed fairly**. That would be pretty cool (laughs). | Formalization |
| Ego 4 | Even if, let's say, a member of the network were to drop out due to illness or something else, it would still run because **everyone is working together and pooling resources**. This makes it more efficient and allows us to compensate for any gaps. | Formalization |
|  | It DEVELOPED as I was introduced to my then new networks – school, neighborhood – OR I was informed that **certain networks existed** in the district or that **there were contact persons in the neighborhood**, and then I gradually (...) went to the meetings. | Bridging |
| Ego 5 | That's what I understand by networking. A specific group of people, or specific individuals, whom I know I can **rely** on and who are there to support projects. | Bonding |
|  | Yes, exactly, building a network like that isn't so easy at the beginning. I would say it's simply because there are **existing structures** (..) because you just kind of join in or participate, for example, working groups or meetings, team meetings or similar. | Formalization |
| Ego 6 | Hmm, the exchange should happen OFTEN, not just once, but **regularly**. So that a **sense of team spirit** develops. | Formalization and Bonding |
|  | This gradually develops more and more, and the (..) **intensity also changes**. The longer you know each other, the more you've worked together, and the more you've tackled similar topics. And if you perhaps share the same problems, the collaboration becomes even more intense. This creates a (..) **sense of group cohesion**. It's a direct result of the collaboration. | Bonding |
| Ego 7 | Then, various stakeholders in the neighborhood tell us um (..) about projects, what's currently happening, and how things are progressing. **They ask who can help with specific problems, who has experience, knowledge, or even personnel resources they can provide**. This is how new connections are formed. | Clustering |
|  | That gave me the chance to benefit from (..) her networks, from her contacts with the parents, because they would then come. **She has the connections, the trust (..) and that benefits me.** | Bridging |
|  | But you don't have a fixed group that comes back every time, and in my experience, that's an important point for collaboration. When there's a **sense of group cohesion**, things, like offers, etc., are more likely to be planned and implemented. (...) | Bonding |
| Ego 8 | Often (..) there are also **subtopics or subgroups** where something is developed collaboratively or towards a common goal, right? Be it a **specific topic or action**, or some need that exists (..) in the neighborhood that needs to be discussed, what has happened. | Clustering |
|  | Another important secondary effect of regular, recurring networking is that it (..) fosters **a sense of cohesion** and belonging. | Bonding |
|  | I have to say, um (..) I think the needs are high. There are many needs and a great deal of will behind them (..) but I think many institutions are **struggling with their own problems**. Be it **staffing** or whatever, **so they're overwhelmed**. And yes, it has happened that contact doesn't work out or only happens much later. | Contextual Barriers |
| Ego 9 | Exactly. Yes, so it usually went like this (...) someone would approach me. They'd say, "We have this and that need," and then **we'd look at who else might be available**. **Who else could contribute expertise or experience?** Who else could we bring on board? And then we'd ask them. "What are you planning? Are you interested and motivated to participate? Do you have any ideas? Would you like to meet up sometime?" And that's how groups were formed. | Clustering |
|  | In some working groups that didn't have a designated person to guide things, I got the feeling that there wasn't much of a sense of responsibility among everyone. And that often led to problems – for example, **ideas that were there were sometimes never implemented**. Because the communication wasn't good enough, **responsibilities weren't clearly defined**, no **deadlines** were set, and you can definitely tell when something like that happens. From what I've learned so far, it seems like someone is needed to coordinate things. | Formalization |
| Ego 10 | Networking works when you **like** each other (..) Absolutely. And if the other person is also **EMPATHETIC** (..) and receptive to the ideas and interests being presented, and shows ways in which they can be realized... | Bonding |
|  | For me, a network is a connection between people who can work together effectively and efficiently **on a certain topic** or on topics where their professional topics overlap. | Clustering |
| Ego 11 | I also joined this network, and there were already things like this before me, and there will be things like this after me, so I don't have to reinvent the wheel. And precisely because of this, I think you can benefit from networks by simply **using existing structures** **and not having to start from scratch every time**. This makes work processes much faster. And (...) that's one of my goals when I work in these networks: that I don't have to build everything from scratch on my own. | Formalization |
|  | I connected with her, **and she then directly introduced me**. She had already been in the neighborhood for, I don't know, a year before me, and she showed me around, took me to the community and social center. **She introduced me to the neighborhood manager, and that's how I got involved.** | Bridging |
|  | and then really show your face and not shine with absence but simply be there and **actively seek contact, build a relationship.** | Bonding |
| Ego 12 | Someone really needs to take on **the organizational tasks**, otherwise it won't work. So, someone needs to send out emails to everyone about when, how, where, and what the meeting is about, who's coming, or who to contact. That's absolutely crucial. Hmm, yes (..) However, I think it's very important that everyone in the network feels equally valued and truly like equal members, and not like the person who moderates is the one (..) who decides everything, and then, well, nobody can say anything against it. Exactly, there has to be a good atmosphere, yes, but moderation is important. | Formalization |
|  | We're planning some activities, **we're gathering needs**, so, uh (..) we've really spent quite a while compiling needs and discussing what's important to us. It's really taken some time, and now we're in a phase of, well, what do we do with all this? | Need-oriented Practice |
|  | The best thing to do is **go there in person**, and that helped me a lot to get to know the neighborhood and see what's available locally, because I need that, so it's also part of my job that I know my way around here so I can guide other people here. | Bonding |
| Ego 13 | Mmm, it was rather intuitive (the search for network partners), but also deliberate with certain institutions, for example the family neighborhood center. Um, because they **have many programs for children and because they also like to support children in our age group**. | Need-oriented Practice |
|  | I think a network is something that you HAVE to **build** and **MAINTAIN**. | Bonding |
|  | So, **who can contribute what, what would be beneficial, don't we need this, and who could procure it for us**? Generally speaking, in this neighborhood, people pay more attention when someone shows interest in ideas and tries to implement them. | Clustering |
| Ego 14 | That wouldn't work without my network, if no one informed the parents or elderly people, or distributed the flyers (...) that a new offer was coming. So (...) (sighs) it's kind of the **BRIDGE between all the activities, because I couldn't do it alone.** | Bridging |
|  | **Trust**, or rather, the **relationship**, is an important aspect of my work. If I put up a flyer somewhere saying, "We're now offering relaxation for mothers," but nobody knows anything about it. They don't know the place, they don't know the person from the office (unintelligible microphone noise) then nobody will go. That means it helps me if people who have perhaps already built trust with some people in the neighborhood say, "Come on over, we have a new program." | Bonding |
|  | And this networking and this use of these resources, the contacts, the premises, the locations, the distribution lists, that was certainly also a goal. **Consultation, event planning, and also identifying the needs in the neighborhood.** What is already known, where are there major problems, where are there gaps in knowledge, where do we need language interpretation? | Need-oriented Practice |

Table S1 Illustrative Interview Quotes and Corresponding Network Strategies
